# Mycobacterium Abscessus Abscess Post-thread Facial Rejuvenation Procedure

**Published:** 2015-04-07

**Authors:** Benny Yau, Clayton Lang, Raja Sawhney

**Affiliations:** Department of Plastic Surgery, Gold Coast Hospital, Southport QLD, Australia

**Keywords:** *Mycobacterium abscessus*, thread facelift, complication, cosmetic tourism

## DESCRIPTION

**Figure 1:**
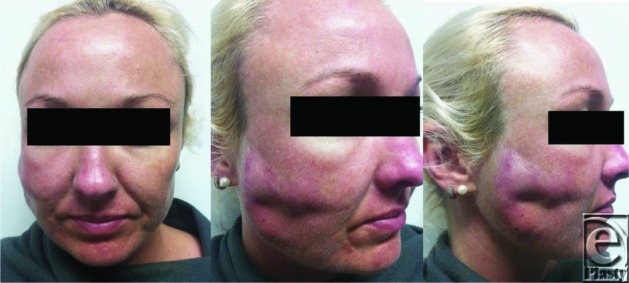
Right facial abscess.

An immunocompetent 33-year-old woman presented to the Gold Coast Hospital, Australia, with an 8× 6-cm abscess of the right cheek, having had a thread face-lift performed in Thailand 6 weeks prior (Fig 1). Imaging revealed linear echogenic foreign bodies within collection. Thirty milliliters of pus was sent for culture and strands of barbed suture were removed from the wound at the time of drainage. Culture identified *Mycobacterium abscessus* and the patient was treated with cefoxitin and clarithromycin.

## QUESTIONS

1. **What is *M abscessus*?**
2. **What is the clinical concern over *M abscessus*?**
3. **What is thread lift facial rejuvenation?**
4. **In which circumstances does *M abscessus* infection occur postsurgery?**

## DISCUSSION

*Mycobacterium abscessus* is a species of the *Mycobacterium* family, which includes the bacterium responsible for tuberculosis and leprosy in humans.^1^ The genus is known for its incredible capacities to resist commonly used antimicrobial agents and survive environmental extremes. *M abscessus* is classified as a rapid-growing *Mycobacterium*, that is, species that can form visible colonies within 7 days.^1^ Recently, a taxonomical change has led to identification of *M abscessus* as the most common rapid-growing *Mycobacterium* in human infection.^2^

*M abscessus* has been identified to have a significant role in skin and soft tissue infections and is a growing concern for clinicians as it displays antibiotic resistance.^3^ Certain factors in the anatomical cell membrane composition enable the bacterium to resist hostile environments and antimicrobial therapy.^3^ The pathogen is mainly associated with clinical infection in immunocompromised patients.^1^

Thread facelift for facial rejuvenation is a relatively new procedure, which employs blind puncture technique to introduce barbed suture material to the subcutaneous pre-SMAS plane to cause gathering of tissues and provides an alternative to conventional surgical facial rejuvenation techniques such as eyebrow/forehead/mid-face lift.^4^ There are multiple thread facelift systems available. A technique that has been examined in literature previously is the Anti-ptosis (Aptos) system, which employs a percutaneous technique using 2-polypropelene suture. Previous literature has reported multiple complications including parotid duct injury, scarring, hematoma, and asymmetry.^5^ This is the first reported case of *M abscessus* facial abscess following this procedure; however, it is uncertain which brand of suture material was used in this case.

Cases of *M abscessus* causing postsurgical wound infection outbreaks have been reported from patients undergoing cosmetic procedures abroad.^6^ The species was isolated to contaminated sinks, instruments, and water in the hospital.^6^ There is growing concern over patient's travelling overseas for cosmetic procedures in an effort to save money. These patients return home with complications of surgery and often do not receive quality postoperative support from the vendor. Frequently the burden falls to the public system to treat the complications of such surgery.^7^

This case highlights the growing role of *M abscessus* as a pathogenic entity. It is emerging as the most pathogenic of the *Mycobacterium* species to humans. It has the ability to be resistant to multiple antibiotics and is therefore quite difficult to eradicate even in an immunocompetent patient.^1^^-^3 The case also highlights the issue of cosmetic tourism and the burden on the public health system as postoperative care is lacking for patients who return home after undergoing a procedure overseas. In these cases, little information is available for the patients preoperatively in regard to the qualifications and standards of the care they are receiving.^7^ Eradication of a *M abscessus* abscess requires prompt multidisciplinary care, with expedient surgical clearance and treatment with appropriate antibiotics under the care of an infectious diseases specialist. *Mycobacterium* should be considered even in immunocompetent patients presenting with infection with a history of travel and surgery.
